# Screening type 2 Diabetes mellitus among Indians using inflammatory biomarkers

**DOI:** 10.6026/973206300200515

**Published:** 2024-05-31

**Authors:** Mohammad Arif, Shreya Nigoskar, Manish Kumar Verma, Ameerul Hasan Amir

**Affiliations:** 1 Department of Biochemistry, Index Medical College &Research Center Indore, Madhya Pradesh, India; 2 Department of Biochemistry, Rajashri Dashrath Autonomous State Medical College Ayodhya, U.P, India; 3 Department of Biochemistry, Autonomous State Medical College, Lalitpur, India

**Keywords:** Inflammatory biomarkers, type 2 diabetes mellitus, pre-diabetes, glycosylated hemoglobin, metabolic health

## Abstract

Diabetes is a metabolic disorder associated with chronic inflammation; pre-diabetes phase promotes to inflammatory mechanism then finally progress to diabetes and its associated complications. Therefore, it is of interest to investigate the changes in inflammatory biomarkers Evidence that inflammatory markers play a role in the development as well as severity of Type 2 diabetes mellitus (T2DM). This study has been designed to decipher the involvement of Tumor Necrosis Factor (TNFα), Interleukin-6 (IL-6), Nesfatin-1 and Blood sugar in the etiopathogenesis of T2DM. This retrospective observational study analyzed patient records from our hospital, focusing on those with diabetes or pre-diabetes. Glycosylated hemoglobin, inflammatory biomarkers, Fasting Blood Glucose, and Post-Prandial Blood Glucose were assessed. SPSS 28 facilitated statistical analysis; utilizing Bivariate Correlation assessed the relationship between inflammatory biomarkers and diabetes status (glycosylated hemoglobin). In the pre-diabetic vs. diabetic groups, significant differences exist in IL-6 (p=0.0344), TNF-α (p=0.041), Nesfatin-1 (p=0.0485), fasting blood glucose (p=0.036), and 2h post-prandial blood glucose (p=0.048). IL6 (AUC=0.729, p<0.001), TNF (AUC=0.761, p<0.001), and Nesfatin1 (AUC=0.892, p<0.001) show moderate discriminative power. PP (AUC=0.992, p<0.001) and hbA1c (AUC=0.993, p<0.001) exhibit excellent discriminatory ability. Correlations: IL6 with TNF (r=0.672, p<0.001) and Nesfatin1 (r=0.542, p<0.001); TNF with Nesfatin1 (r=0.591, p<0.001), hbA1c (r=0.683, p<0.001), and PP (r=0.367, p<0.001); Nesfatin1 with PP (r=0.594, p<0.001) and hbA1c (r=0.800, p<0.001). Age has a negative correlation with hbA1c (r=-0.119, p=0.086). Thus, data shows a significant association between inflammatory markers, blood glucose levels, and the progression from pre-diabetes to diabetes.

## Background:

**Figure 1:**
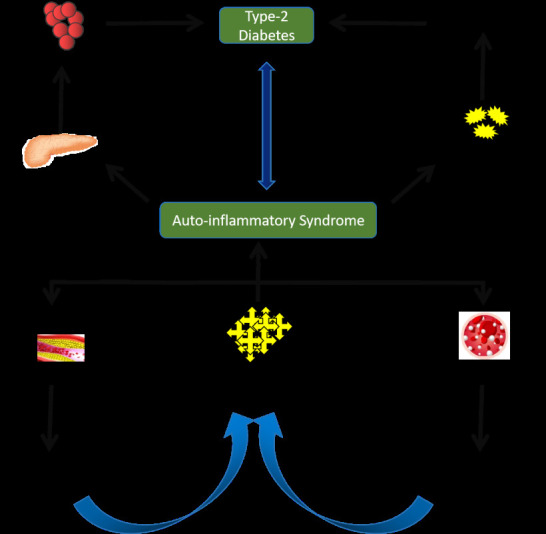
Relationship between inflammation and type-2 diabetes

A prediabetic individual is considered between the normoglycemia and diabetes, whose Fasting Blood Glucose typically, ranges from 100 to 120 mg/dL [1]. Around 86 million individuals in the United States, or one in three, had pre-diabetes in 2012. Individuals are mostly unaware of the diagnosis, which can go up to 90% of the patients [1, 2]. A Survey by the International Diabetes Federation has shown that about 318 million individuals globally were anticipated to have impaired glucose tolerance (IGT) in 2015. It is estimated that it will increase to 482 million by 2040. Evidences have shown that developing diabetes has significant correlation with increasing age, increasing insulin resistance (IR), inadequate insulin production, and other risk elements [2]. Every year, it is estimated that the incidence of diabetes is about 5-10%. There are several preventive steps that can be followed by an individual for minimize the risk of developing diabetes including lifestyle changes, diet, and physical activities [3, 4]. Clinical studies have shown that the combined therapy of drug and lifestyle modification can return the most significant results and prevent diabetes. Since 1990, the number of diabetics has been in rise and currently, 8.8% of the adult population found to have diabetes with more male preponderance. It is also expected that the number of diabetics will rise to 700 million by 2045 [1-3]. Population studies have shown that the highest diabetic population is in India, China, and USA and this is expected to continue to rise even in 2045, according to International Diabetes Federation (IDF). There is an increasing need of screening programme, to prevent population wide diabetic complications and increasing the economic burden throughout the countries [4,5]. Peripheral IR, reduced incretin release, insulin secretion anomalies, glucotoxicity, lipotoxicity, reduced β-cell function, oxidative stress, and inflammation mass all contribute to β-cell failure are among the many variables that contribute to the development of prediabetes. IGT, or isolated impaired fasting glucose, is the classification used to describe prediabetes [6]. There is disagreement about the appropriate limits for glycated haemoglobin (HbA1c) and glucose in the diagnosis of dysglycemic conditions since the World Health Organization and the American Diabetes Association, among others, have different recommendations. We will go over a few more indicators that are used to forecast the likelihood of developing diabetes [7]. A pronounced inflammatory state is a characteristic of both IR and prediabetes. Acute-phase reactant and inflammatory cytokine biochemical indicators are raised with the outset of type 2 diabetes and may rise even more as the illness progresses. Certain indicators such fibrinogen, C-reactive protein, and white blood cell count (CRP) have been investigated as possible indicators of type 2 diabetes developments, as seen in studies like Atherosclerosis Risk and Communities [8]. There are suggestions put forward as the mechanisms that come into play in causing diabetes. It starts from the genetic level and dietary and lifestyle factors including increase consumption of glucose than its requirements, leading to hyperglycemia. Increase consumption of Free Fatty Acid (FFA) and Low-Density Lipoprotein (LDL) cholesterol which leads to dyslipidaemia. This causes the inflammatory mediators to increase in the serum. Along with this, there is developing beta-cell dysfunction leading to its apoptosis and eventually decreasing the insulin secretion. This leads to type-2 diabetes. Increased inflammatory mediator's results in auto-inflammatory syndrome, which in turn, results in inflammation in peripheral tissue and development of insulin resistance. Both the decreased secretion of insulin and developing insulin resistance, results in type-2 diabetes [5-7, 9]. Figure 1 shows the summarized mechanism of the above explanation.

CRP is the most well researched inflammatory marker associated with CVD, and its application in medicine is still developing. CRP is the principal indicator for the initial response and is mostly produced by hepatic biosynthesis that is dependent on IL-6. Numerous studies have shown that people in T2DM and IR had larger amounts of both IL-6 and CRP [9]. 1,625 participants were tracked for 5.2 years in a multicentre trial called as Insulin Resistance Atherosclerosis trial (IRAS). 132 People who developed diabetes during follow-up were classified as prediabetes. Insulin-sensitive non-diabetics and prediabetic individuals did not have higher CRP levels than individuals who were both insulin resistance and prediabetic [10]. These variations were believed to be somewhat caused by variations in body weight. Subclinical inflammation is not exclusively linked to hyperglycemia since those IR and prediabetes did not result in hyperglycemia [11].

Further indicators of inflammation and immunology that may be clinically significant for the development of the illness and consequences unique to individual organs in diabetes include the fibrinogen and white blood cell count [12]. Furthermore, leucocytosis might indicate illness of the heart valves. As a result, early detection of high-risk individuals might stop the development of CVD or at least slow its course. It has been demonstrated that in Pima Indians, a lot of white blood cells is predictive of declining insulin action, insulin secretion function, regarding the onset of diabetes type 2. Four Hundred because fibrinogen alters blood viscosity, platelet aggregation, and fibrin production, it may have a role in atherosclerosis. Moreover, fibrinogen influences fibrinolysis and coagulation activation, which may promote the development of plaque [13].

In western nations, type-2 diabetes has a substantial cardiovascular risk which occurs due to a combination of chronic low-grade inflammation and other risk factors [14]. Accordingly, it has been shown by epidemiological studies that elevated inflammatory mediators in plasma, such CRP, IL-6, and TNF-α are observed in individuals having metabolic syndrome. This is manifested by the clinical features of type 2 diabetes. The concentrations of other molecules, such Monocyte Chemo attractant Protein-1 (MCP1), Lipoprotein-Associated Phospholipase A2 (LP-PLA2), and transforming growth factors (also known as tumour growth factor) TGF-β1, are also higher in T2D patients [15]. Obesity and central adiposity might result from a hereditary tendency connected to an excessive consumption of calories and inactivity. Subsequently, this might lead to malfunctioning of adipose tissue, the infiltration of macrophages, and an increased secretion of cytokines like TNF-α and IL-6. Extended increases in these markers are linked to endothelial dysfunction, skeletal muscle insulin resistance, and hepatic CRP release. Furthermore, endothelium and macrophages produce IL-6 in response to hyperglycemia, which may exacerbate insulin release and signaling cascades. This implies that enhancing glycemic management may lessen the inflammatory reaction, hence strengthening the association between inflammation and disruptions in the metabolism of glucose [16].

## Methods:

## Research Design:

This current retrospective observational study has obtained the patients records who visited our hospital with either diabetes or pre-diabetes as diagnosed by two consultants blinded to each other. The patients were tested for Glycosylated hemoglobin, other inflammatory biomarkers, and Fasting Blood Glucose and Post-Prandial Blood glucose. The observation was made regarding the level of the inflammatory markers with that of Glycosylated hemoglobin. The research included 210 patients, comprising of 105 patients each in pre-diabetic group and Diabetes group. The author measured HbA1c values (mean% ± SD) were analyzed for gender-specific and overall categories within each group. The pre-diabetic and diabetes groups used the same approach for collecting and analyzing these variables. Various indicators were evaluated between pre-diabetic and diabetes groups. The parameters measured were IL-6, TNF-α, nesfatin-1, fasting blood glucose, and 2-hour postprandial blood glucose. To establish the significance of differences between the two groups, p-values were determined for each parameter. IL-6, TNF-α, nesfatin-1, fasting blood glucose, and 2-hour postprandial blood sugar levels were examined across pre-diabetic and diabetic groups to identify disease progression biomarkers. All metrics were collected and analyzed using the same way to assure dependability.

## Inclusion Criteria:

[1] The patient is diagnosed with diabetes or pre-diabetes based on the fasting blood glucose and glycosylated hemoglobin.

[2] Patients with type 2 Diabetes Mellitus (according to ADA criteria for diabetes)i

[3] The patient who visited our hospital outpatient department.

[4] The patient who gave consent to share the results of all the parameters

## Exclusion Criteria:

[1] Patients with any systemic disease e.g. asthma chronic obstructive pulmonary disease (COPD), I malignancies, Sexually Transmitted Diseases, cardiovascular disease.

[2] Patients with Type 1 diabetes mellitus.

## Statistical analysis:

The study has used SPSS 27 for effective analysis. The continuous data has been expressed as Mean±SD deviation while the discrete data were expressed frequency and its percentage. The graphs were plotted in MS Excel. The graphs also showed the variation of the biomarkers with that of the level of Glycosylated hemoglobin. The authors employed Bivariate Correlation to statistically analyze the correlation of the levels of inflammatory biomarkers to that increasing status of diabetes measured as glycosylated hemoglobin. ROC was plotted from glucose measurements, HbA1c and measurements of other inflammatory mediators for assessing the predictive capability. The study also conducted bivariate correlation between the factors using SPSS 27. The level of significance was P<0.05.

## Ethical approval:

The Ethical Committee of the hospital approved the study method before collection of the data was started by the authors.

## Results:

Table 1 presents baseline characteristics of patients categorized into pre-diabetic and diabetic groups in the study, focusing on various parameters such as age, 2-hour postprandial glucose levels, sex distribution, and glycosylated hemoglobin (HbA1c) levels. Notably, significant differences emerge between the two groups, particularly regarding 2-hour postprandial glucose and HbA1c levels. The mean 2-hour postprandial glucose levels are substantially higher in the diabetic group (292.095 ± 60.90) compared to the pre-diabetic group (173.41 ± 17.42), with a highly significant p-value of less than 0.001. This finding underscores the clear distinction between the glucose control states of the two groups, with the diabetic group exhibiting markedly elevated postprandial glucose levels indicative of uncontrolled diabetes. Similarly, the HbA1c levels demonstrate a statistically significant difference between the pre-diabetic (6.09 ± 0.27%) and diabetic (6.86 ± 0.23%) groups, with a p-value of less than 0.001. This result suggests a substantial disparity in long-term glycemic control between the two groups, with the diabetic cohort showing significantly higher HbA1c levels, reflective of poorer overall blood sugar management. However, no significant differences are observed in age distribution or sex composition between the pre-diabetic and diabetic groups, as indicated by p-values of 0.120 and 0.240, respectively. Overall, these findings highlight the importance of assessing not only fasting glucose levels but also postprandial glucose and HbA1c levels in differentiating between pre-diabetic and diabetic states, emphasizing the clinical relevance of these parameters in diagnosing and managing diabetes.

**Figure 2:**
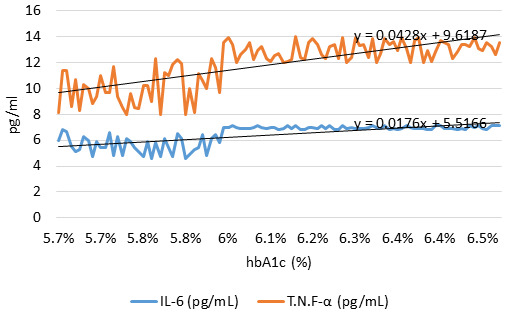
The relationship between glycosylated hemoglobin (HbA1c) levels and IL-6 and TNF-α

The variation between glycosylated hemoglobin (HbA1c) and IL-6 and TNF- levels is seen in Figure 2. As HbA1c grows from 5.7% to 6.5%, there appears to be a pattern of growing IL-6 and TNF-α level. Increased inflammatory activity, as measured by elevated levels of IL-6 and TNF-, may be linked to hyperglycemia, as measured by HbA1c. If you are trying to figure out how to treat a condition like diabetes, where chronic inflammation is a major issue, keeping an eye on these signs could be a big help.

**Figure 3:**
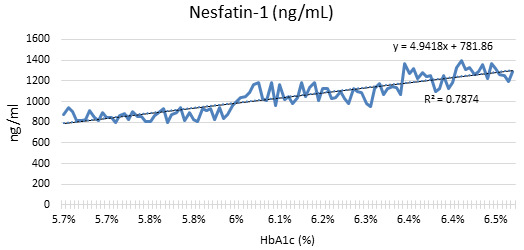
The relationship between glycosylated hemoglobin (HbA1c) levels with that of Nesfatin-1

Figure 3 shows that Nesfatin-1 concentrations are inversely proportional to levels of glycosylated hemoglobin (HbA1c). Nesfatin-1 levels are increasing as HbA1c rises from 5.7% to 6.5%. This may indicate a correlation between HbA1c (a measure of blood sugar levels) and Nesfatin-1 secretion. Glucose metabolism may affect Nesfatin-1, a hormone known for its function in appetite and energy control. This finding provides new information about the dynamic relationship between glucose homeostasis and appetite-related hormones, highlighting the complexity of the interplay between the two. Monitoring Nesfatin-1 alongside HbA1c could provide significant information in evaluating metabolic health.

The levels of inflammatory markers and blood glucose in the pre-diabetic and diabetic populations are shown in Table 2. Compared to the pre-diabetic group, those with diabetes have greater levels of IL-6 (7.020.20 pg/mL) and TNF- (13.561.17 pg/mL) in their blood. There is also an increase in the appetite-regulating hormone nesfatin-1 (1290.10 ng/mL) from pre-diabetes (1043.77169.60 ng/mL) to diabetes. As inflammation, glucose dysregulation, and diabetes progression are all linked, it is not surprising that fasting and 2h post-prandial blood glucose levels are significantly higher in the diabetes group (152.23 mg/dL and 292.09 mg/dL) than in the pre-diabetes group (112.79 mg/dL and 173.41 mg/dL).

**Figure 4:**
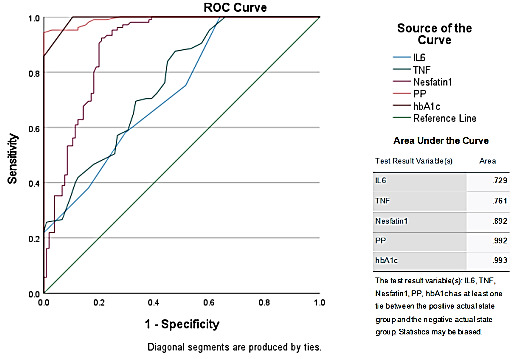
ROC showing the Area under the Curve for each inflammatory mediator, 2-hour Post Prandial Glucose and HbA1c

Figure 4 provides the Area under the Curve (AUC) values for various test result variables, including IL6, TNF, Nesfatin1, PP, and hbA1c. The AUC values for the different variables are as follows: IL6 (0.729), TNF (0.761), Nesfatin1 (0.892), PP (0.992), and hbA1c (0.993). These values suggest varying degrees of discriminative power among the variables. PP and hbA1c demonstrate the highest discriminatory ability, with AUC values close to 1, indicating excellent performance in distinguishing between the target outcomes. Nesfatin1 also shows a high AUC value, indicating strong discriminatory power. IL6 and TNF exhibit lower AUC values compared to the other variables but still signify reasonable discriminative ability. Overall, these AUC values provide insights into the effectiveness of each test result variable in distinguishing between relevant outcomes, which can be crucial in diagnostic or predictive contexts, such as in healthcare or research settings.

Table 4 provides a comprehensive overview of the area under the curve (AUC) for different test result variables, including IL6, TNF, Nesfatin1, PP, and hbA1c. Each variable's AUC, standard error, significance level, and 95% confidence interval are detailed. For IL6, the AUC of 0.729 suggests a moderate predictive ability for distinguishing between positive and negative outcomes, supported by a significant p-value (p < 0.001) and a confidence interval ranging from 0.663 to 0.796. Similarly, TNF exhibits a comparable AUC of 0.761, indicating moderate predictive ability with a significant p-value and confidence interval of 0.698 to 0.824. Nesfatin1 stands out with an AUC of 0.892, signifying high predictive ability, supported by a significant p-value and a confidence interval from 0.847 to 0.938. Notably, PP and hbA1c demonstrate exceptional predictive power, with AUCs of 0.992 and 0.993, respectively. Their highly significant p-values (p < 0.001) and narrow confidence intervals underscore their robust discrimination between positive and negative outcomes, ranging from 0.984 to 1.000 for PP and from 0.986 to 0.999 for hbA1c. Despite potential bias due to ties between positive and negative groups, these variables maintain their strong predictive capabilities, as evidenced by the compelling statistical results.

Table 3 presents the table presents the findings of bivariate correlation analysis between various factors: IL6, TNF, Nesfatin1, PP, hbA1c, and age, along with their respective P-values indicating statistical significance. IL6 correlates positively with TNF (r = 0.672, p < 0.001) and Nesfatin1 (r = 0.542, p < 0.001), suggesting strong positive relationships. Similarly, IL6 also correlates positively with hbA1c (r = 0.625, p < 0.001) and PP (r = 0.350, p < 0.001), although to a lesser extent. TNF shows strong positive correlations with Nesfatin1 (r = 0.591, p < 0.001), hbA1c (r = 0.683, p < 0.001), and PP (r = 0.367, p < 0.001). Nesfatin1 correlates positively with PP (r = 0.594, p < 0.001) and hbA1c (r = 0.800, p < 0.001), indicating strong positive relationships. However, its correlation with age (r = -0.056, p = 0.423) is not statistically significant. PP correlates positively with hbA1c (r = 0.726, p < 0.001), while its correlation with age (r = -0.071, p = 0.309) is not statistically significant. The correlation between hbA1c and age is negative (r = -0.119, p = 0.086), indicating that as age increases; hbA1c levels tend to decrease slightly. This correlation is statistically significant. Finally, age shows weak correlations with all other factors, with most correlations being statistically non-significant. The only significant correlation is with hbA1c (r = -0.119, p = 0.086), indicating a negative relationship between age and hbA1c levels.

## Discussion:

One major cause of type 2 diabetes mellitus (T2DM) constitutes a grave worldwide health concern is obesity. Chronic low-grade inflammation connected to active illness is partly caused by the innate immune system becoming activated. Insulin homeostasis and action are disrupted in obesity due to the primary anabolic cascades being blocked by Interleukin-1β, interleukin-6, and tumour necrosis factor-α are examples of pro-inflammatory cytokines released because of this activation [17]. Acute-phase reactants such haptoglobin, serum amyloid-A, plasminogen activator inhibitor-1, and C-reactive protein are also produced in response to cytokines. The initial (pre-clinical) stages of type 2 diabetes are marked by enhanced production Comprising acute-phase proteins and pro-inflammatory cytokines (inflammatory network), which show a graded rise with the advancement of the illness. According to available data, studying inflammatory networks may help identify novel biomarkers that might be used to identify how risk factors from the environment and genes interact during the onset of diabetes type 2 [18]. Along with the ability to predict disease incidence beyond already monitored risk variables, like regular clinical chemical profiling, lifestyle evaluation, and family history, these biomarkers hold great promise for improving public health. In addition, inflammatory indicators might be useful in assessing new preventative approaches, especially those related to micronutrients [19]. Subclinical inflammation is seen in patients having type 2 diabetes, and almost all markers of systemic inflammation. Elevated quantities of inflammatory indicators in the bloodstream are indicative of a systemic and subclinical inflammatory process to investigate the degree of subclinical inflammatory in those suffering from type 2 diabetes and to determine if glycemic control and indicators of inflammation. Among those who have type 2 diabetes, a substantial correlation exists between inflammation and glycemic control, which shows is a significant factor in the pathophysiology of diabetes [20]. Type 2 diabetes affects the immunological and inflammatory systems. The purpose of the research was to investigate the immunological and inflammatory response in a sizable sample of individuals with diabetes and prediabetes who were typical of the population. The onset and course of type 2 diabetes are correlated with different profiles of inflammatory and immunological biomarkers. Distinguishing from the early preclinical and clinical stages of the disease, as well as its consequences and development, is made possible by markers in inflammation and immunity [21]. An increase in inflammatory markers is one of the factors contributing to the greater cardiovascular risk associated with diabetes mellitus type 2 (T2D). In those who have type 2 diabetes and poor glycemic control, the aim of the research was to determine the connection between inflammatory biomarkers and the glycemic management and low-density lipoprotein (LDL) sub fraction phenotype. People suffering from type 2 diabetes (T2D) exhibit elevated levels of inflammatory biomarkers, particularly in those with obesity and LDL subtype B. Enhancing glycemic management lowers TGF-β1 levels, potentially partially accounting for its properties [22]. An increasing body of research suggests that inflammatory indicators contribute to the onset and progression of Type 2 Diabetes Mellitus (T2DM). The goal of the research is to ascertain how Tumour Necrosis Factor (TNFα), Type 2 diabetes has several etiopathogenic factors, including Interleukin-6 (IL-6), Interleukin-10 (IL-10), and C - reactive protein (CRP) [23]. The results of an investigation demonstrated a strong correlation between type 2 diabetics and TNFα, IL-6, CRP, and IL-10, receiving care at SMHS Hospital who is of Kashmiri origin. In turn, this suggests that cytokines might be useful indicators of the onset of type 2 diabetes [24]. Diabetes type 2 (T2DM) is a significant worldwide health issue. Pre-diabetes mellitus, or pre-DM, is a stage of the illness that occurs before T2DM and is frequently misdiagnosed. In a pre-DM mouse model caused, the purpose of a high-fat diet (HFD) was to find new pre-DM biomarkers. Male C57BL/6J mice received a standard high-fat chow diet for duration of 12 weeks [25]. Samples of liver and serum were separated according to a time-dependent protocol. Cytokine array analysis was used to undertake a semi-quantitative evaluation of secretory cytokines. Thirteen cytokines were chosen for additional examination according to changes in their levels of expression between the pre-DM and T2DM phases [26]. As pre-DM progressed to T2DM, Mice on a high-fat diet resulted in weight gain, elevated blood lipids, insulin, glucose, and liver enzymes. When mice were fed a diet rich in fat, the transcription of inflammatory and lipogenic genes was increased. Measuring protein levels indicated that the pre-DM had increased mRNA expression of the following proteins: soluble 1, Fc the receptor, IgG, is low-affinity adiponectin that binds sugar and galactose protein, as well as growth arrest-specific are examples of adhesion molecules in the bloodstream [27].

## Conclusion:

Data shows that a significant association between inflammatory markers, blood glucose levels, and the progression from pre-diabetes to diabetes. Individuals with diabetes exhibit elevated levels of IL-6, TNF-α, and nesfatin-1, indicating a link between inflammation, glucose dysregulation, and diabetes advancement. The inverse relationship observed between nesfatin-1 and glycosylated hemoglobin (HbA1c) suggests a potential correlation, shedding light on the intricate interplay between glucose metabolism and appetite-related hormones. Monitoring Nesfatin-1 alongside HbA1c could offer valuable insights into metabolic health evaluation. Additionally, the correlation between HbA1c and IL-6/TNF-α levels emphasizes the role of chronic inflammation in diabetes, suggesting the importance of these markers in therapeutic considerations for this condition. While the study elucidates the association between inflammatory biomarkers and diabetes progression, further exploration is warranted to elucidate the mechanistic links and identify predictive markers for disease onset. Future studies could investigate longitudinal changes in inflammatory profiles in prediabetic individuals, exploring the predictive value of inflammatory markers in identifying those at high risk of developing diabetes. Integrating advanced molecular techniques and machine learning algorithms could enhance the predictive accuracy of inflammatory markers in diabetes risk assessment, enabling personalized preventive strategies. Additionally, investigating the modulation of inflammatory pathways through lifestyle interventions or pharmacotherapy may unveil novel therapeutic targets for diabetes prevention and management.

## Ethics approval and consent to participate:

The study was approved by the Institute Ethics Committee, Index Medical College, Hospital & Research Center, Indore, Questionnaire, and informed consent was obtained from all the patients.

## Consent for publication:

All authors have declared that no financial support was received from any organization for the submitted work.

## Funding:

No funding was received for this research.

## Author contributions:

Concept and Design: Mohammad. Arif, Shreya Nigoskar, Manish Kumar Verma

Performed the experiments: Mohammad Arif

Acquisition, analysis and interpretation of data: Mohammad Arif, Ameerul Hasan Amir

Drafting of the manuscript: Mohammad Arif

Critical review: Mohammad Arif, Shreya Nigoskar, Manish Kumar Verma

Validation & Supervision: Shreya Nigoskar, Manish Kumar Verma

## Figures and Tables

**Table 1:** Baseline characteristics of the patients in each group of this study

**Parameters**	**Pre-diabetic group n=105**	**Diabetes group n=105**	**P-value**
Age (years; mean±sd)	54.31±10.28	52.03±10.81	0.12
2-hour Post Prandial Glucose	173.41±17.42	292.095±60.90	<0.001
**Sex**			
Male	67 (63.80%)	75 (71.42%)	0.24
Female	38 (36.19%)	30 (28.57%)	
Glycosylated hemoglobin (HbA1c) ; mean%±sd			
Total	6.09±0.27%	6.86±0.23	<0.001
Male	6.1±0.27	6.85±0.24	
Female	6.09±0.27%	6.9±2.3	

**Table 2:** Findings of inflammatory markers and blood glucose level in each case group

**Parameters**	**Pre-diabetic group**	**Diabetes group**	**P-value**
IL-6 (pg/mL)	6.45±0.77	7.02±0.20	0.03
TNF-α (pg/mL)	11.88±1.74	13.56±1.17	0.04
Nesfatin-1 (ng/mL)	1043.77±169.60	1290.10±71.56	0.04
Fasting blood glucose [mg/dL]	112.79±6.87	152.23±12.76	0.03
2h post prandial blood glucose [mg/dL]	173.41±17.42	292.095±60.90	0.04
**Correlation is significant at the 0.05 level.

**Table 3:** Findings of ROC analysis for case group in each biochemical factor

**Test Result Variable(s)**	**Area**	**Std. Error**	**Asymptotic Sig. (P-value)**	**Asymptotic 95% Confidence Interval**	
				**Lower Bound**	**Upper Bound**
IL6	0.729	0.034	0	0.663	0.796
TNF	0.761	0.032	0	0.698	0.824
Nesfatin1	0.892	0.023	0	0.847	0.938
PP	0.992	0.004	0	0.984	1
HbA1c	0.993	0.003	0	0.986	0.999

**Table 4:** Findings of Bivariate Correlation between the factors

		**IL-6**	**TNF- α**	**Nesfatin1**	**PP**	**HbA1c**	**Age**
IL-6	Correlation Coefficient	1	.672**	.542**	.350**	.625**	0.064
	Sig. (2-tailed)	.	0.001	0.001	0.001	0.001	0.357
	N	210	210	210	210	210	210
TNF- α	Correlation Coefficient	.672**	1	.591**	.367**	.683**	-0.029
	Sig. (2-tailed)	0.001	.	0.001	0.001	0.001	0.671
	N	210	210	210	210	210	210
Nesfatin1	Correlation Coefficient	.542**	.591**	1	.594**	.800**	-0.056
	Sig. (2-tailed)	0.001	0.001	.	0.001	0.001	0.423
	N	210	210	210	210	210	210
PP	Correlation Coefficient	.350**	.367**	.594**	1	.726**	-0.071
0.001	Sig. (2-tailed)	0.001	0.001	0.001	.	0	0.309
	N	210	210	210	210	210	210
HbA1c	Correlation Coefficient	.625**	.683**	.800**	.726**	1	-0.119
	Sig. (2-tailed)	0.001	0.001	0.001	0.001	.	0.086
	N	210	210	210	210	210	210
Age	Correlation Coefficient	0.064	-0.029	-0.056	-0.071	-0.119	1
	Sig. (2-tailed)	0.357	0.671	0.423	0.309	0.086	.
	N	210	210	210	210	210	210
**Correlation is significant at the 0.01 level (2-tailed).
